# Reduced Interference and Serial Dependency Effects for Naming in Older but Not Younger Adults after 1 Hz rTMS of Right Pars Triangularis

**DOI:** 10.1162/nol_a_00063

**Published:** 2022-03-17

**Authors:** Jonathan H. Drucker, Charles M. Epstein, Keith M. McGregor, Kyle Hortman, Kaundinya S. Gopinath, Bruce Crosson

**Affiliations:** VA Rehabilitation Research & Development Center for Visual and Neurocognitive Rehabilitation, Decatur, GA, USA; Department of Neurology, Emory University, Atlanta, GA, USA; Aptima, Inc., Woburn, MA, USA; Department of Radiology, Emory University, Atlanta, GA, USA; Department of Psychology, Georgia State University, Atlanta, GA, USA

**Keywords:** aging, language, inhibition, neuromodulation, repetitive transcranial magnetic stimulation, Broca’s area

## Abstract

1 Hz repetitive transcranial magnetic stimulation (rTMS) was used to decrease excitability of right pars triangularis (R PTr) to determine whether increased R PTr activity during picture naming in older adults hampers word finding. We hypothesized that decreasing R PTr excitability would reduce interference with word finding, facilitating faster picture naming. 15 older and 16 younger adults received two rTMS sessions. In one, speech onset latencies for picture naming were measured after both sham and active R PTr stimulation. In the other session, sham and active stimulation of a control region, right pars opercularis (R POp), were administered before picture naming. Order of active vs. sham stimulation within session was counterbalanced. Younger adults showed no significant effects of stimulation. In older adults, a trend indicated that participants named pictures more quickly after active than sham R PTr stimulation. However, older adults also showed longer responses during R PTr than R POp sham stimulation. When order of active vs. sham stimulation was modeled, older adults receiving active stimulation first had significantly faster responding after active than sham R PTr stimulation and significantly faster responding after R PTr than R POp stimulation, consistent with experimental hypotheses. However, older adults receiving sham stimulation first showed no significant differences between conditions. Findings are best understood, based on previous studies, when the interaction between the excitatory effects of picture naming and the inhibitory effects of 1 Hz rTMS on R PTr is considered. Implications regarding right frontal activity in older adults and for design of future experiments are discussed.

## INTRODUCTION

While the left hemisphere is normally dominant for language, older adults show right (R) frontal activity during language production that is not present in younger adults. It has been suggested that activity during language production in aging plays a compensatory role, assisting a declining left hemisphere with language tasks such as word finding (e.g., Cabeza, 2001, 2002; Cabeza et al., 2004; Dolcos et al., 2002). Increased R frontal activity has been shown for older relative to younger adults both for picture naming (Berlingeri et al., 2013; Fridriksson et al., 2006; Hoyau et al., 2017; Wierenga et al., 2008) and for category-member generation (Meinzer et al., 2009, 2012; Persson et al., 2004).

Usually this increased R frontal activity for older adults is located in R pars triangularis (PTr), the anterior portion of the R hemisphere homologue for Broca’s area, though not always exclusively so. Wierenga et al. (2008) found that for poorer performing older adults, picture naming was negatively correlated with blood oxygen level-dependent (BOLD) activity in R PTr. More importantly, Meinzer et al. (2009, 2012) found for all older adults that accuracy of category-member generation was negatively correlated with R PTr BOLD activity during this task. In other words, the greater the R PTr activity, the poorer word-finding performance was. These findings raise the possibility that R PTr activity in older adults interferes with word finding. Indeed, Nocera et al. (2017) found that after a three-month aerobic exercise regimen, lower post-exercise intervention activity in R inferior frontal cortex during category-member generation was associated with increased category-member generation accuracy and increased efficiency in oxygen utilization from pre- to post-exercise intervention. The association of lower R frontal activity post intervention with an increased accuracy in category member generation across the intervention is highly suggestive that R frontal activity impedes word finding in older adults.

A more direct way to determine if R PTr activity is interfering with word finding, however, is to use non-invasive brain stimulation (NIBS) and observe the effects on a word-finding task. Repetitive transcranial magnetic stimulation (rTMS) and transcranial direct current stimulation (tDCS) are common forms of NIBS. While rTMS has advantages, tDCS has been used more commonly to study frontal language functions in older adults and clinical populations (Fridriksson et al., 2011). In tDCS, a constant, low amplitude current (1–2 mA) is passed between an anode and a cathode. Generally, the anode is thought to increase cortical excitability beneath it while the cathode is thought to decrease cortical excitability.

Holland et al. (2011) found that anodal tDCS of left (L) inferior frontal cortex during picture naming, with the cathode over R frontopolar cortex, decreased verbal reaction times compared to sham tDCS for older adults. There were no younger controls in this study. Meinzer et al. (2013) found that anodal tDCS of the L inferior frontal gyrus (IFG), with the cathode over R supraorbital cortex, improved accuracy of category-member generation in older adults compared to sham tDCS. For bilateral stimulation of the IFG (anode over L IFG, cathode over R IFG), Lifshitz Ben-Basat and Mashal (2017) found faster reaction times during active than sham tDCS in older participants, but only in the sham-first group and not in the active-first group (active and sham conditions were separated by at least two or three days). There was no young control group.

From the standpoint of our question about R PTr, there are potential limitations to tDCS studies. Two studies (Holland et al. 2011; Meinzer et al. 2013) investigated only L inferior frontal anodal stimulation, and one study (Lifshitz Ben-Basat & Mashal, 2017) used anodal stimulation in L and cathodal in R IFG. Thus, none of the three studies performed isolated cathodal stimulation of the R IFG to decrease its excitability and determine effects on word finding. Further, two of the studies (Holland et al., 2011; Lifshitz Ben-Basat & Mashal, 2017) did not include a young control group to which the results of the findings for older participants could be referenced. Finally, given the tDCS electrodes and their configuration, isolation of PTr stimulation from stimulation of other IFG components would have been extraordinarily difficult, even if attempts to isolate R IFG stimulation had been made (Dmochowski et al., 2011). Hence, the question of whether increased R PTr activity interferes with word finding remains an open question.

The current study was designed to address this issue. We elected to use 1 Hz repetitive transcranial magnetic stimulation (rTMS), which decreases cortical excitability, to deal with some of the limitations to tDCS studies. rTMS induces rapid current changes that are limited to a relatively small and well-defined brain region; i.e., it has greater spatial specificity than tDCS. When paired with neuronavigation guided by structural magnetic resonance images, rTMS affords greater precision in stimulation of target cortical regions. Our study also included a young control group to which we could reference findings from older adults. Further, based on previous work in aphasic patients and neurotypical adults (Naeser et al., 2005, 2011; Ren et al., 2014), we included a control region, R pars opercularis (POp), for R PTr and performed active and sham rTMS on both R PTr and R POp. Since R POp is just posterior to R PTr in the IFG, even high-definition tDCS (i.e., using multiple channels on the scalp to selectively target specific brain regions) could not provide enough spatial resolution to separate stimulation of PTr from that of POp. We hypothesized that active 1 Hz rTMS of R PTr would reduce reaction times for picture naming in older adults relative to sham stimulation of R PTr and relative to active stimulation of R POp, because R PTr activity interferes with the word-finding functions of L PTr, and that reducing the excitability of R PTr will reduce its activity, thereby reducing its interference. In other words, according to this hypothesis, this effect would be achieved because the decreased excitability of R PTr would reduce its interference with picture naming. Because young adults do not show R PTr activity during word finding (Meinzer et al., 2009, 2012; Wierenga et al., 2008), we did not expect to see changes in picture-naming reaction times after 1 Hz rTMS in young adults.

## MATERIALS AND METHODS

### Participants

Participants were 15 older adults (8 female) who were 65 to 79 years of age (mean = 70.67 years, *SD* = 4.91) with at least a high school education (12–19 years, mean = 15.71 years, *SD* = 2.05) and 16 younger adults (8 female) who were 20 to 32 years of age (mean = 25.25, *SD* = 3.21) with at least a high school education (16–21 years, mean = 16.79 years, *SD* = 1.58). (Due to a recording error, years of education were not available for one older and two younger adults.) Participants were recruited through flyers posted on the Emory University campus, in local libraries, and in retirement communities, or by advertisements in local news papers. All participants had English as a first language and were right-handed. The Montreal Cognitive Assessment (MoCA; Nasreddine et al., 2005) was used as a cognitive screening tool. A cut-off score of 24 or higher was used for inclusion, consistent with norms for the region in which the study took place (Luis et al., 2009). Further, participants had to be free from risks for magnetic resonance imaging (MRI) scanning (e.g., cardiac or other pacemakers, ferromagnetic implants not anchored to bone, significant claustrophobia) and chronic conditions that could affect cognitive functions (e.g., traumatic brain injury, epilepsy or family history of epilepsy, Parkinson’s disease, Alzheimer’s disease, stroke, heart failure, kidney failure, malaria). Subjects could not be on antiseizure medications or other medications that might reduce responsiveness to rTMS, or on medications that might reduce seizure thresholds (e.g., bupropion, varenicline, chlorpromazine, theophylline). Because the IFG lies below the temporalis muscle, which could be stimulated by TMS pulses, persons with temporomandibular joint disorder were excluded from participation. Because exercise has been shown to affect language functions and brain activity in older adults (Zlatar et al., 2013; Nocera et al., 2015, 2017, 2020), and we wanted to measure the effects of age without the confound of exercise effects, persons who regularly performed moderate to high levels of exercise for at least 45 min on at least three days per week were excluded. Participants gave written informed consent in accordance with procedures established by the Emory University/Atlanta VA Medical Center Institutional Review Board, consistent with the Declaration of Helsinki. They were paid $50 per session for each of three sessions.

### Procedures

#### Screening and cognitive testing

Respondents to advertisements and flyers underwent a brief telephone screening to ensure that they met criteria for the study. Prior to MRI scanning and after informed consent procedures during the first session, subjects participated in a brief cognitive assessment. As noted above, the MoCA (Nasreddine et al., 2005) was administered as a cognitive screening tool. Potential participants achieving a score of 24 points or higher proceeded to MRI scanning and subsequently were scheduled for their two rTMS sessions. To further characterize our sample, the California Verbal Learning Test (Delis et al., 2000) and the Boston Naming Test (Kaplan et al., 2001) were administered prior to scanning.

#### Magnetic resonance imaging

**Figure 1.**
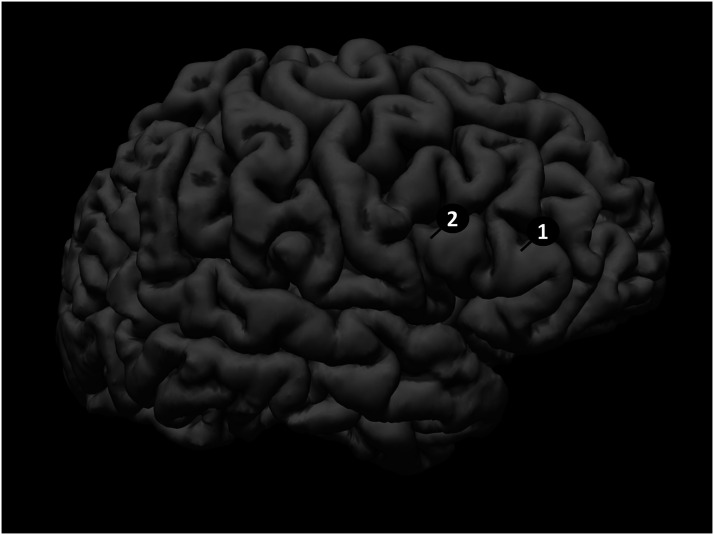
Stimulation sites for rTMS in R IFG. “1” represents a typical R PTr stimulation site. “2” represents an example of a R POp site.

After consenting and cognitive testing, the younger and older adults participated in an MRI scanning session. A T1-weighted (3 dimensional MP-RAGE) structural MRI was acquired to assist in image-guided rTMS (TR = 2,300 ms TE = 2.89 ms, flip angle = 8 degrees, spatial resolution = 1 × 1 × 1 mm, matrix = 256 × 256, 176 sagittal slices). Sites for rTMS were selected as follows. Anatomic landmarks were derived from the location of R PTr activity differences between old and young participants during a previous picture-naming study (Wierenga et al., 2008), providing optimal separation of stimulation between the two areas (PTr, POp) (Figure 1). Specifically, for R PTr a line was drawn from the intersection of the anterior horizontal ramus (AHR) and the anterior ascending ramus (AAR) of the Sylvian fissure to the inferior frontal sulcus (IFS) at roughly a 45 degree angle to the AHR. The stimulation site was about half way up this line from the vertex. For R POp, the stimulation site was in the superior portion of the area (posterior to the AAR and near the IFS), in most instances providing at a least 1.5 cm separation between stimulation sites.

#### rTMS and picture-naming sessions

Experimental procedures were modeled after those of Naeser et al. (2011), a study of low frequency (1 Hz) rTMS of PTr, POp, and control regions in aphasic persons and cognitively neurotypical adults. For Naeser et al., two or fewer stimulations occurred within a single session, with at least 30 min between stimulations. In the current study, younger and older adults participated in two rTMS sessions at least a day apart: one session for active and sham PTr stimulation and one session for active and sham POp stimulation. The order of PTr and POp sessions was counterbalanced across participants within each group. Across participants, the order of active and sham stimulation within sessions also was counterbalanced, but each participant had the same order for active vs. sham rTMS for both sessions. At least 30 min elapsed between active and sham stimulation for each area.

One Hz rTMS was delivered via a MagVenture MagPro X100 stimulator and a Magventure Cool-B65 A/P liquid-cooled figure of eight coil. Participants received 600 pulses over 10 min (1 Hz) at 90% of resting motor threshold. R PTr and R POp were localized as described above using a Rogue Research Brainsight 2 neuronavigation image guidance system. Two electrodes for sham stimulation were placed prior to rTMS sessions approximately 1 cm below the hairline and 2 cm apart on the right side of the forehead. For active stimulation, the coil was oriented with the junction vertical (handle pointing downward) to limit stimulation primarily to the target structure, with the stimulating side of the coil held directly over the scalp. Position of the coil relative to the target was continuously monitored using the Brainsight neuronavigation system so that real-time adjustments could be made by the operator to keep the coil over the target site in R PTr or R POp. For sham stimulation, the inert side of the coil was held over the scalp near the target location. The rTMS protocol (1 Hz stimulation for 10 min) was run so that the audible clicks of the magnetic pulses were present during both active and sham stimulation, but stimulation was delivered to the brain only during active rTMS. Electrical stimulation on the forehead was used during active and sham stimulation to enhance the similarity of sensations between the conditions. At the start of both active and sham sessions, electrical pulses were delivered through the surface electrodes in synchrony with the audible clicks of the coil and were adjusted to low levels so that the stimulation was not painful to the participants.

#### Naming task

Target names for high imageability items were selected from the University of Western Australia Psycholinguistic Database (https://websites.psychology.uwa.edu.au/school/MRCDatabase/mrc2.html). Four lists of 30 items were composed of 15 medium frequency (4–20 occurrences/million) and 15 low frequency (less than 4 occurrences per million) words, and each list contained similar numbers of living and nonliving items. Lists also were counterbalanced such that there were no significant differences between lists for frequency of words in the English language, number of letters, number of syllables (1–4), familiarity, imageability, or concreteness. Four sets of 30 colored pictures corresponding to the target words from the four lists were selected from freely licensed internet databases. Immediately after each of the four rTMS conditions (PTr active, PTr sham, POp active, POp sham), participants sat in front of a Dell Latitude E6420 laptop computer on which one of the four picture sets was presented. A different picture set was presented for each of the conditions. Picture sets were counterbalanced across the conditions such that each picture set appeared an equal number of times in each condition. To avoid participants getting into a rhythm that could bias response latencies, presentation was paced by the experimenter, and a 500 ms pre-stimulus fixation preceded every trial. A new picture was presented after response to the previous picture was completed. Vocal responses were recorded for off-line scoring. The first word uttered in response to a picture was scored for correctness; non-word utterances were ignored for scoring purposes. Plausible alternative responses to the target picture names were allowed. Only correct responses were used in the reaction time analysis; responses more than 3 *SD*s greater than an individual participant’s mean reaction time were excluded from analysis. Once a correct response was identified, the speech onset latency (SOL; i.e., reaction time) for the response was calculated using an automated MATLAB script written specifically for the current study, removing any bias from derivation of verbal reaction times.

SOLs were analyzed using a linear mixed effects model that controlled for systematic variance related to the specific items, thereby reducing error variance. The effects of interest were age (young vs. old, a between-subjects effect), site of stimulation (PTr vs. POp, a within-subjects effect), kind of stimulation (active vs. sham, a within-subjects effect), and order of stimulation (active first vs. sham first, a between-subjects effect). Since order effects were not a part of experimental hypotheses, main experimental hypotheses were first assessed without respect to order. As noted in the Introduction, our a priori hypothesis was that active stimulation of R PTr would reduce reaction times for picture naming in older adults relative to sham stimulation by decreasing its excitability, with no corresponding effect in younger participants. A further a priori hypothesis was that older participants would name pictures more quickly after active PTr than active POp stimulation, with no corresponding effect in younger participants. Subsequently, post hoc analyses assessed if effects for order of stimulation within sessions (active first vs. sham first) interacted with the effects of age (younger vs. older) and/or type of rTMS (active vs sham).

## RESULTS

### A Priori Hypotheses

**Figure 2.**
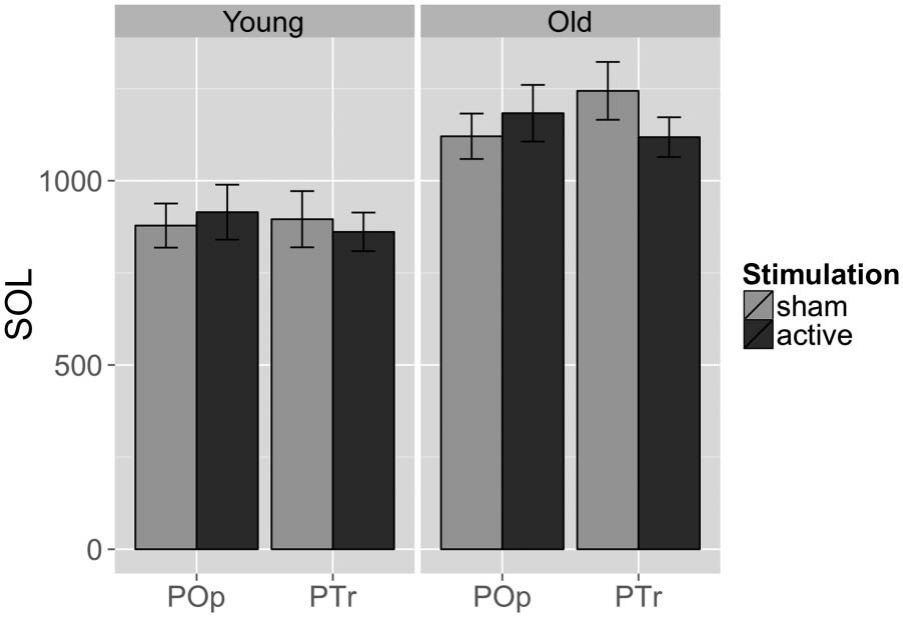
Speech onset latencies (SOLs) for younger and older participants after 1 Hz active or sham rTMS of pars opercularis (POp) and of pars triangularis (PTr). The lighter gray bars represent sham stimulation, and the darker gray bars represent active rTMS. For older participants (right), there was a marginal difference between active vs. sham stimulation for PTr (sham SOL minus active SOL = 135 ms, *p* = 0.0664). However, the sham condition for PTr was slower than that for POp (PTr minus POp = 145 ms, *p* = 0.0143). There were no significance differences for the younger group (left).

A priori hypotheses were tested using the structure of a 2 target (PTr vs. Pop) × 2 stimulation (active vs. sham) Analysis of Variance (ANOVA), removing variance in SOLs related to the specific target words from error variance using a linear mixed model. The first a priori hypothesis, derived from previous functional MRI studies (Berlingeri et al., 2013; Meinzer et al., 2009, 2012; Wierenga et al., 2008), was that for R PTr, active low-frequency rTMS would lead to faster naming than sham stimulation in older but not younger adults, with no such effects for our control region (R POp). Figure 2 shows mean SOLs for active vs. sham stimulation in each of the target brain areas (R PTr vs. R POp) for older vs. younger adults. For PTr in older participants, the mean difference for active vs. sham of −135 ms was marginally significant (*p* = 0.0664). The corresponding mean difference of 79 ms for R PTr in younger adults was not significant (*p* = 0.2648). For older participants, the effect for active vs. sham stimulation of R POp (69 ms) was not significant (*p* = 0.1725), and the effect for R POp in younger adults (29 ms) was not significant (*p* = 0.5518).

Our other hypothesis was that active stimulation of R PTr would yield faster reaction times than stimulation of the control region (R POp) in older participants. This hypothesis was not confirmed (R POp minus R PTr = 59 ms, *p* = 0.3690), with similar results in younger participants (R POp minus R PTr = 54 ms, *p* = 0.3394). Finally, inspection of Figure 2 indicated a large difference between the sham conditions for PTr vs. POp in the older adults. While this comparison was not planned, it might have explanatory value for our other findings, and this analysis indicated the difference was substantial (R PTr sham minus R POp sham = 145 ms, *p* = 0.0143).

A perplexing aspect of our data was the difference in sham conditions for R PTr vs. R POp stimulation. By definition, the sham conditions had no active stimulation of the cortex and thus should not produce a measurable behavioral effect. The fact that sham stimulation of R PTr produced significantly longer SOLs than sham stimulation of R POp indicates that something was affecting the cortex in one sham condition that was not affecting it in the other. Two facets of our data led us to ask whether within-session order effects for active vs. sham stimulation in our study might account for the difference in SOLs between the R PTr and the R POp sham conditions.

First, while our within-session time span between active and sham rTMS stimulation (30 min) was similar to other rTMS studies of language (e.g., Naeser et al., 2011) at the time our study was planned, reports of order effects in older participants, even with days between sessions, began to emerge for tDCS (e.g., Lifshitz Ben-Basat & Mashal, 2017) and rTMS (e.g., James et al., 2017). Indeed, in the motor system, Siebner et al. (2004) had previously shown that the effects of tDCS can last at least 35–45 min. Hence, when active stimulation is done first with sham 30 min later, the effects of active stimulation may still be present when sham stimulation is given that would not be present when sham stimulation was done first.

The second consideration indicating that the order effects were worth examining was that based on the findings of Wierenga et al. (2008), our picture-naming tasks involved repetitive excitation (30 events) of R PTr over a few minutes. Lang et al. (2004) showed that preconditioning weak excitatory stimulation with stimulation that reduces cortical excitability tends to enhance the effects of a weak excitatory stimulus 20–30 min after the latter, and Siebner et al. (2004) showed that preconditioning stimulation that is normally inhibitory evokes a weak excitatory stimulation that, in turn, lessens effects of the inhibitory stimulation 25–35 min after preconditioning. Hence, these studies raise the possibility that our picture-naming tasks were interacting with our 1 Hz (inhibitory) r TMS. As we discuss in detail later (in the Discussion section), these sequences in Lang’s and Seibner’s studies have analogues in our study. For purposes of studying order effects, the important point here is that the interaction of 1 Hz rTMS of R PTr with the excitatory effects on R PTr of picture naming might produce order effects in older adults affecting results of the stimulation condition (active or sham) that was administered second within sessions. In other words, there were no experimental events that could precondition the stimulation condition (active or sham) that was administered first, but the picture naming or active rTMS administered during the first stimulation condition could precondition the events that followed it to affect picture-naming SOLs in the second condition. Hence, taking these possibilities into consideration, within session order was added to the model to determine if it could further explain our findings.

### Adding Order of Stimulation to the Analysis Model

Hence, we repeated the above linear mixed model ANOVA adding order of stimulation within session (active first vs. sham first) as a third independent variable. Since significant effects for the above a priori comparisons were found for the older but not the younger group, we focused these analyses on the older group, but also performed a parallel analysis in the younger group to determine whether order effects could have contributed to any lack of findings in the younger subjects. For the older group, there was a significant 3-way interaction (*p* = 0.0426) of Cortical Target (PTr vs. POp) × Stimulation (Active vs. Sham) × Order within Sessions (active-first vs. sham-first). Hence, we next determined whether there were Target × Stimulation interactions at the different levels of order within sessions. For the active-first group, the Target × Stimulation interaction was significant (*p* = 0.0018), but for the sham-first group, this interaction was not significant (*p* = 0.7962). Given this finding, we next looked at pairwise comparisons representing stimulation effects at the different targets (R PTr vs. R POp), as well as the target effects at the different types of stimulation, for the active-first older group.

**Figure 3.**
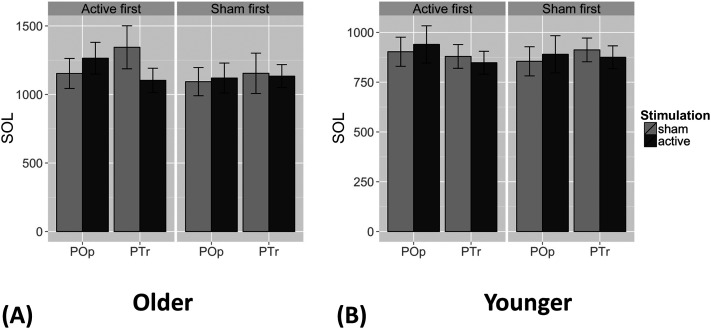
(A) Speech onset latency (SOL) reactions times for the active-first (left) and sham-first (right) conditions for the older adults. Light gray bars represent sham stimulation, and dark gray bars represent the active stimulation. For the active-first group, participants named pictures significantly faster for active than sham rTMS in PTr but not in POp. Older participants also named pictures faster after active PTr stimulation than after active POp stimulation. However, they also named pictures faster after sham POp stimulation than after sham PTr stimulation. The unexpected difference between sham conditions is explained in the Discussion section. (B) SOLs for the active-first (left) and sham-first (right) conditions for younger adults. Younger participants named pictures marginally faster after active PTr stimulation than after the active POp condition, but no other comparisons approached significance.

For the older participants, the active-first effects (Figure 3A) are an amplified version of the results when order of stimulation was not modeled, specifically for the R PTr conditions: The R PTr active minus sham difference (−291 ms) was significant (*p* = 0.005), consistent with our a priori hypothesis. Further, the R PTr active minus the R POp active difference of −183 ms was significant for active-first participants (*p* = 0.038), which also is consistent with our a priori hypothesis. The 226 ms difference between the sham conditions (R PTr sham vs. R POp sham) also was significant (*p* = 0.008). The 26 ms difference for R POp active minus sham was not significant (*p* = 0.708). There were no significant interactions for the younger participants.

### Accuracy

Average accuracy of picture-naming performance was 99.54% correct for younger participants (average error percentage = 0.47%, *SD* = 0.74%) and 98.10% correct for older participants (average error percentage = 1.90%, *SD* = 3.26%). Twelve of the 15 older subjects had error rates within the distribution limits of the younger participants (0–3 errors for all four of the word lists, 120 items). The other three older participants had 4, 6, and 15 errors for all four of the word lists, accounting for 73.53% of the total errors for the older group. Table 1 shows the error rates for the younger and older participants per 30-word list. Given the high accuracy rates for both groups (all but 1 younger and 3 older participants averaged ≤0.5 errors per list), it was not surprising that further analysis of errors with respect to stimulation conditions and sites of stimulation did not yield significant differences characterizing the performance of either group.

**Table 1.** Number of participants with different error rates per list for younger and older participants

**Group**	**Error rates per 30-word list**			
	***0 Errors / List***	***0.25 Errors / List***	***0.5 Errors / List***	***≥0.5 Errors / List***
***Younger***	10	4	1	1
***Older***	5	5	2	3

## DISCUSSION

The main hypothesis for this study was that decreasing excitability of R PTr in older adults would improve the efficiency of picture naming because this R PTr activity interferes with word finding. If suppression of R PTr activity via 1 Hz rTMS in the older group resulted in faster picture naming, it would provide clear evidence that R PTr activity had been *interfering* with word retrieval, as opposed to *compensating* for diminished function. Our study was designed to enable us to test this hypothesis against two control conditions: (1) sham (as opposed to active) stimulation, and (2) a control target in R POp, a brain region that previous literature (Naeser et al., 2011; Wierenga et al., 2008) indicated would not respond to 1 Hz rTMS. Results support our hypotheses with a caveat that warrants detailed discussion and suggests future avenues of research.

Namely, despite precedent in the literature for a 30-min washout period between conditions, our results suggested that 30 min between the active and sham conditions was insufficient, and that the inhibitory effects of rTMS (active-first condition) or excitatory effects of picture naming (sham condition) interacted with other experimental events that followed in the session, confounding the effects of stimulation (active vs. sham rTMS) and/or target (R PTr vs. R POp) (Lang et al., 2004; Siebner et al., 2004). To explore this possible interaction, we stratified our sample by the order in which they received active vs. sham stimulation within session and repeated our analysis. This was statistically and practically feasible because the groups were well-counterbalanced. In so doing, we discovered that the hypothesized effects in one older group (active-first) were obscured by the lack of any effects in the other group (sham-first).

The remainder of the discussion deals with the following: (1) We interpret the results of the R PTr vs. the R POp stimulation conditions in the active-first group within the context of our experimental hypothesis. (2) We explain how experimental manipulations in the condition administered first (active or sham) interacted with subsequent experimental conditions to affect the results of the condition administered second in older adults. (3) The implications of our findings for future experimental design as well as the broader implications for rehabilitation are discussed. Finally, a brief summary is offered.

### R PTr vs. R POp in Active-First Group

We examined the possibility that order of stimulation within session (active first vs. sham first) interacted with type of stimulation (active vs. sham) and target of stimulation (R PTr vs. R POp), emphasizing older adults. This order of stimulation within sessions had been counterbalanced across participants, with half in each group receiving active rTMS first on each of the two visits, and half receiving sham rTMS first on each visit. When the temporal order of active vs. sham rTMS was included in our statistical model, we found convincing support for our experimental hypotheses in the active-first group (Figure 3). This evidence had been partially obscured in our primary analysis by the lack of an effect in the sham-first group. Further, there was a significant difference in the sham conditions for R PTr vs. R POp, with the sham condition for R PTr having significantly longer SOLs (i.e., slower responding) than for R POp in the active-first group, calling into question the validity of the comparisons of the active and sham conditions. Thus, we rely on the comparison of R PTr and R POp active stimulation in the active-first group to address our hypothesis regarding R PTr activity during picture naming.

After 1 Hz rTMS of R PTr in the older active-first group, SOLs for picture naming were faster than after 1 Hz rTMS of the control area, R POp. This finding supports the idea that decreasing the excitability of (i.e., inhibiting) R PTr is removing its interference with picture naming. It also contradicts the concept that R PTr activity is a compensatory mechanism that aids picture naming because inhibiting such a compensatory mechanism would make picture naming less efficient (i.e., slower), the opposite effect from our findings. One might ask: Why does R PTr activity increase during picture naming if it is not compensatory? The work of McGregor et al. (2013, 2018) shows an analogous situation in the motor system to that in the language system. Specifically, R motor cortex (M1) shows decreased activity in younger adults during R hand movements that changes to increased activity in sedentary older (middle aged) adults (McGregor et al., 2013), similar to Wierenga et al.’s (2008) picture-naming findings in R PTr. (Recall that adults in the present study also were sedentary.) The increased R M1 activity in older adults is accompanied by poorer performance in speeded and skilled motor activities of the R hand (McGregor et al., 2013). Further, decreased R hand motor skills in sedentary older adults is associated with decreased interhemispheric inhibition of R M1 after single-pulse TMS stimulation of L M1 (McGregor et al., 2013), indicating that reduced interhemispheric inhibition in older adults might account for the poorer motor performance. Since the subjects in McGregor’s and Wierenga’s experiments were right-handed, we can assume left-hemisphere dominance for motor and language functions in these experiments. These parallels suggest that the relationship between interhemispheric inhibition and word-finding efficiency should be investigated in older adults.

### Serial Dependency Effects and Active vs. Sham Comparisons in Older Adults

The reader will remember that for the active-first group, sham stimulation for the R PTr session showed significantly slower SOLs than sham stimulation for the R POp session. Theoretically, this should not happen because sham stimulation should have no effects on performance. Further, the reader will recall that there were no differences in SOLs after the active vs. the sham condition for the sham-first group of older adults. It was asserted earlier that these two findings, which were contrary to our predictions, could be explained by interactions between the final picture-naming measure in the stimulation paradigm that was administered second and the experimental events that preceded it. Indeed, the veracity of the above interpretation of R PTr vs. R POp stimulation effects depends to some degree on our ability to explain these unexpected findings.

#### Why SOLs in the sham condition for R PTr are increased

**Figure 4.**
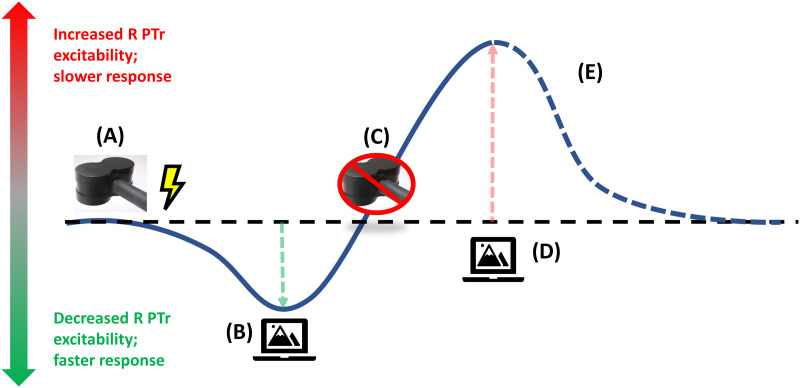
Reversed polarity effects during sham PTr condition for participants receiving active rTMS first. (A) Active 1 Hz rTMS is applied to R PTr. (B) First picture-naming task. A moderate decrease in R PTR excitability leads to faster (i.e., shorter) speech onset latency. However, the act of word retrieval, potentiated by the preceding rTMS, begins to strongly increase excitability of R PTr. (C) Sham rTMS. Even though no rTMS is delivered, the excitability effects just described are accompanied by an increase in R PTr excitability. (D) Second picture-naming task. A strong increase in R PTr excitability leads to slower (i.e., longer) speech onset latency. (E) It is uncertain how long the increased R PTr excitability might last after the second picture-naming task, so this phase is depicted by a dashed line.

To understand why the SOLs for sham stimulation in the R PTr session of the active-first group were longer than those for sham stimulation in the R POp (control) session, we must understand the order of events that produce the serial dependencies for the picture-naming measure in the sham condition. Picture naming for sham stimulation in the active-first group was preceded first by active 1 Hz rTMS, which has the effect of inhibiting R PTr. This stimulation was followed by repetitive picture naming which tends to excite R PTr in older adults (Wierenga et al., 2008). The reader may recall from the study of Lang et al. (2004) that inhibitory stimulation followed by weak excitatory stimulation (20 s of 5 Hz rTMS) significantly enhances the effects of excitatory stimulation, producing enhanced excitatory effects above that of receiving only sham stimulation at 20–30 min post excitatory stimulation, similar to the timeframe of the current experiment. Thus, the increased excitatory effects of picture naming that are preconditioned by 1 Hz rTMS should increase the interference effects of R PTr activity evoked by picture naming after sham stimulation, which, in turn, would cause increased SOLs for the measurement taken after sham stimulation. The effects of this sequence of events are illustrated in Figure 4. Further, it must be emphasized that this explanation requires that R PTr interferes with picture naming, providing further evidence for our hypothesis that R PTr in older adults interferes with picture naming.

#### Why there is no difference between active and sham stimulation in the sham-first group

To understand the serial dependency for the active condition in the sham-first group, we must again review the order of events. The first active event for this group is the picture-naming measurement after sham stimulation, which has an excitatory impact on R PTr. This is followed later by 1 Hz (inhibitory) rTMS. A study speaking to the order of events for the sham-first group is that of Siebner et al. (2004). They performed 10 min of anodal tDCS stimulation (which increases cortical excitability), followed 10 min later by 15 min of 1 Hz rTMS. Siebner et al. found that greater excitability in response to anodal tDCS leads to greater suppression of cortical excitability after 1 Hz rTMS, and that *weaker response to anodal tDCS results in weaker suppression of excitability after 1 Hz rTMS*. Hence, if the excitatory stimulation afforded by 30 picture-naming trials is weak in terms of its excitatory after-effects (because of a small number of trials, or the spacing between trials (~2–4 s), etc.), then its preconditioning could weaken the effects of 1 Hz rTMS, thereby negating the rTMS effects. Such a sequence, then, could lead to a lack of difference between the sham and active 1 Hz rTMS for the sham-first older group. (It should be remembered that the effects on the sham trial of the active-first group discussed in the preceding paragraph require only weak excitatory stimulation; Lang et al., 2004).

### Implications of Serial Dependency Effects

The current report has introduced the idea that there may be serial dependency between repeated behaviors and NIBS techniques that may introduce unexpected effects on subsequent behavior. In the following paragraphs, we will discuss two important issues regarding these serial dependencies. The first issue is how to avoid the kind of confounds that interaction between NIBS and repetitive behavior introduce into experimental designs and their interpretation. Hopefully, this discussion can be of some help avoiding such interpretative complications in the future. Second, NIBS interventions are receiving a great deal of attention in the rehabilitation literature (Crosson et al., 2019). Hence, interactions between NIBS and behavioral treatments could invoke unexpected consequences (positive or negative) during rehabilitation studies or treatments. These two topics are covered below.

#### Avoiding serial dependencies between NIBS and repetitive behavior

An important implication of the preceding discussion is that repetitive picture naming (or other repetitive behaviors) should not be viewed solely as inert measurements. They may have a weak excitatory effect that interacts with preceding or subsequent brain stimulation or another episode of picture naming. This hypothesis is itself worth putting to further test because a knowledge of the excitatory effects of repetitive picture naming could affect the design of future experiments in which NIBS techniques are used to test neurocognitive theories. The key consideration in such experiments is how to mitigate the interactive effects of NIBS and picture naming. One obvious remedy for the order effects described above is to put more time between active inhibitory, active excitatory, and/or sham conditions with repetitive behavioral measurements of their effects. The 30 min between conditions of the current study obviously was not enough, but how much more time is enough? In the tDCS neuromodulation literature, it is common to put a week between sessions, consistent with recent recommendations by Woods et al. (2016) for avoiding long-term neuroplasticity effects of tDCS. For example, in their study of the effects of tDCS on word retrieval, Meinzer et al. (2013) had at least a week between active and sham tDCS sessions. Further, in the one study with only two to three days between sessions (Lifshitz Ben-Basat & Mashal, 2017) there was also an order effect. Based upon these considerations, the recommendation of a week between different active and/or sham sessions to avoid accumulation of long-term effects seems appropriate for any NIBS technique.

#### Implications for rehabilitation

We have discussed ways in which repeated behavioral activation of cortex and NIBS of the same cortex might interact to potentiate or negate the effects of NIBS. Potentially, there are numerous ways in which such serial dependencies might be studied and eventually applied in rehabilitation. Here, we mention one example: It has become common for studies to use anodal stimulation of language-eloquent dominant-hemisphere cortices combined with language training for aphasia in rehabilitation studies (see Crosson et al., 2019, for a review, or Fridriksson et al., 2018, for a more recent example). Lang et al. (2004) showed that anodal stimulation of M1 followed by a weak excitatory stimulation of the same cortex resulted in a reversal of the excitatory effects of anodal tDCS so that they became inhibitory effects that began somewhere between 10–20 min after the initial tDCS. It has not been uncommon to begin language training during tDCS and to continue it after tDCS has been ended. But, if language training has a weak excitatory effect on the language-eloquent cortex that is stimulated, then, could continuing language training much past the end of tDCS be counter-productive because it eventually leads to inhibitory effects on the stimulated language-eloquent cortex that is needed for successful language training? Further, could differences in whether or how long the behavioral component of therapy continued after tDCS account for some of the differences in outcomes between combined tDCS and behavioral treatment that were noted by Crosson et al. (2019). This analysis suggests that it would be worth investigating the best time after tDCS to stop language training to achieve optimal results.

### Conclusions

The current study was designed to test the proposition that R PTr activity in older adults interferes with picture naming. Specifically, 1 Hz rTMS, shown to reduce cortical excitability, was administered to R PTr immediately before SOLs to picture naming were measured. It was hypothesized that this low-frequency rTMS would suppress the interference of R PTr on picture naming, leading to faster SOLs. When within-session order effects were modeled, findings indicated that there were serial dependencies between picture naming and 1 Hz rTMS that invalidated comparisons with sham stimulation for r PTr. Nonetheless, in the active-first group of older adults, comparison of active R PTr stimulation with active stimulation of a control area, R POp, showed faster SOLs for R PTr, indicating that R PTr activity in older adults interferes with word finding. Further, when taken in the context of the work of Lang et al. (2004), the lengthening of SOLs after sham stimulation in the active-first group also indicates R PTr activity interferes with word finding in older adults. No evidence from this study supports the idea that R PTr activity provides compensatory support of word finding in older adults. Serial dependencies between behavioral activation and NIBS, such as those found in the current study, can be avoided in future studies of the cognitive effects of NIBS by allowing the effects of NIBS to dissipate over one week before performing other active or sham NIBS conditions. Finally, the implications of interactions between repetitive behavioral manipulations and NIBS for rehabilitation should be studied.

## ACKNOWLEDGMENTS

Work on this study was supported by the following grants: Grant # I21 RX00099401 (Bruce Crosson), Senior Research Career Scientist Award # B6364-L (Bruce Crosson), and Career Development Award – Level 2 # IK2 RX000956 (Keith M. McGregor), all from the United States Department of Veterans Affairs Rehabilitation Research and Development Service. The views expressed in this work do not necessarily reflect those of the United States Government or the Department of Veterans Affairs. All authors contributed significantly to the production of this work.

## FUNDING INFORMATION

Bruce A. Crosson, U.S. Department of Veterans Affairs (https://dx.doi.org/10.13039/100000738), Award ID: I21 RX00099401. Bruce A. Crosson, U.S. Department of Veterans Affairs (https://dx.doi.org/10.13039/100000738), Award ID: B6364-L. Keith M. McGregor, U.S. Department of Veterans Affairs (https://dx.doi.org/10.13039/100000738), Award ID: IK2 RX000956.

## AUTHOR CONTRIBUTIONS

**Jonathan H. Drucker**: Conceptualization: Supporting; Data curation: Lead; Formal analysis: Lead; Investigation: Lead; Methodology: Equal; Project administration: Supporting; Software: Lead; Visualization: Equal; Writing – original draft: Equal; Writing – review & editing: Equal. **Charles M. Epstein**: Conceptualization: Supporting; Methodology: Equal; Resources: Supporting; Supervision: Supporting; Writing – original draft: Supporting; Writing – review & editing: Equal. **Keith M. McGregor**: Conceptualization: Equal; Data curation: Equal; Funding acquisition: Equal; Investigation: Supporting; Methodology: Supporting; Supervision: Supporting; Writing – review & editing: Equal. **Kyle Hortman**: Data curation: Supporting. **Kaundinya S. Gopinath**: Methodology: Supporting. **Bruce Crosson**: Conceptualization: Lead; Formal analysis: Supporting; Funding acquisition: Lead; Methodology: Equal; Project administration: Lead; Resources: Lead; Supervision: Lead; Visualization: Supporting; Writing – original draft: Equal; Writing – review & editing: Lead.

## COMPETING INTERESTS

Charles M. Epstein received royalties from Neuronetics, Inc., which manufactures transcranial magnetic stimulators. No Neuronetics equipment was used in this study.

## TECHNICAL TERMS

Pars triangularis (PTr): A structure in the anterior portion of inferior frontal gyrus.
Repetitive transcranial magnetic stimulation (rTMS): In rTMS, repeated pulses of magnetic energy penetrate the skull, inducing a current that stimulates underlying cortex.
Transcranial direct current stimulation (tDCS): In tDCS, direct current stimulation is applied to the brain through electrodes placed on the scalp.
Pars opercularis: A structure occupying the posterior portion of the inferior frontal gyrus.
1 Hz rTMS: When rTMS pulses are delivered at a 1/second rate, excitability of cortex is decreased, making it more difficult to activate.
